# Reply to the letter to the editor by Manzoli, Acuti Martelucci and Flacco (doi: 10.17179/excli2025-9176)

**DOI:** 10.17179/excli2026-9442

**Published:** 2026-04-15

**Authors:** Marco Alessandria, Franco Berrino, Giovanni M. Malatesta, Alberto Donzelli

**Affiliations:** 1 Department of Theoretical and Applied Sciences, eCampus University, 22060 Novedrate, Italy; 2 Department of Life Sciences and Systems Biology, University of Turin, 10123 Turin, Italy; 3 (past) Department of Predictive and Preventive Medicine, Fondazione IRCCS Istituto Nazionale dei Tumori, 20133 Milan, Italy; 4 Scientific Committee of the Foundation "Allineare Sanità e Salute", 51100 Pistoia, Italy; 5 Independent Medical-Scientific Commission, Foundation "Allineare Sanità e Salute", 20131 Milan, Italy

## ⁯⁯⁯

We appreciate the journal's policy of providing space for scientific debate on the articles it publishes. We are writing this further letter to correct three statements by Manzoli et al. (2025[11]) regarding our Comment (Malatesta et al., 2025[10]) and our previous articles, which distort their meaning.

1. Manzoli et al. (2025[11]) state: *“...which, however, produced either crude rates* (Berrino et al., 2023[3]) *or multivariable models that were inexplicably not adjusted for age* (Alessandria et al., 2024[2])*.”* This could give readers the impression that Berrino et al. and Alessandria et al. lacked transparency, and we believe this clarification is necessary.

In the first cited text, Berrino et al. (2023[3]), the limitations and intentions are clearly stated: *“It must be clear that these computations are merely an exercise to illustrate the direction of the bias and do not pretend to provide true estimates of the effect.”* Despite this, we are convinced that the research findings of both Flacco et al. (2023[5]) and Acuti Martellucci et al. (2025[1]), which attribute a significant benefit to mRNA products for all causes of death (including non-COVID causes), are questionable, as no biologically plausible reason could explain a therapeutic effect of mRNA products for all causes of death. Therefore, any estimate showing such an association should raise suspicion of serious bias, requiring thorough investigation by the scientific community.

Instead, the second text cited by Manzoli et al. (i.e. Alessandria et al., 2024[2]), carried out the correction for age and explicitly stated that *“Additional information collected to adjust the estimated HRs included nine covariates: sex, AGE, hypertension, diabetes, chronic obstructive pulmonary disease (COPD), cardiovascular disease (CVD), kidney diseases, cancer, and infection (individuals infected by SARS-CoV-2),”* and that furthermore *“With the aim to verify the validity of assumption of the model, Schoenfeld's test was used considering both the value of the global test and each covariate, so we can use the appropriate stratification if the assumptions of the model were not satisfied.”* Applying stratification implicitly allows the HR to be controlled for covariates (such as age) that do not satisfy the model's proportionality assumptions. Stratification, for age or other covariates, is necessary to obtain reliable HR values when those covariates do not meet the model assumptions (Kleinbaum and Klein, 2012[9]).

2. Manzoli et al. continue by stating that *“... our use of Cox models, which aligned time zero, eligibility, and treatment, accounted for the different duration of follow-up of exposed and unexposed individuals (Acuti Martellucci et al., 2025*[1]*).”* However, in the section describing the use of the Cox model, there are no statements supporting the procedure. In fact, simply adopting Cox models in itself does not align time zero, eligibility, and treatment assignment unless the dataset design does so. Conversely, what is written in their “Follow-up” section appears to support the hypothesis of inadequately addressed immortal time, which could explain a significant bias in their results.

3. Furthermore, Manzoli et al. add: *“Also, […] correcting for the immortal time bias is debatable as the vaccinated individuals would appear twice with issues of intra-cluster variance (Hernán et al., 2016*[7]*).”* However, the method we used is the one proposed by Mantel-Byar, considered the gold standard and recognized as a procedure that provides unbiased estimates of associations (Weberpals et al., 2018[13]; Mi et al., 2016[12]). The Mantel-Byar method is based on time-dependent information, where each subject contributes unexposed and exposed person-time based on the patient's exact exposure status at each point during follow-up (i.e., the time a subject spends in each of the two categories - first unvaccinated, then vaccinated - is precisely separated. When the subject transitions from one category to the other, person-time calculation in the first category stops, censorship occurs, and person-time calculation restarts with the subject in the new category. Therefore, it is the time a person spends within a category that is considered, not the person - Hanley and Foster, 2014[6]). We have no record that this has any relevance to the paper by Hernán et al. (2016[7]) cited by Manzoli et al., which, moreover, does not mention any intra-cluster variance issue.

We reiterate that we are also convinced that healthy-vaccinee bias is among the causes of the implausible mortality results reported by Acuti Martellucci et al. (2025[1]), as they themselves acknowledge. This bias is probably even greater than they claimed and persists much longer than Chemaitelly et al. (2025[4]) estimated, as demonstrated by the example of Jackson et al. (2006[8]), cited in our letter, among many other possible examples. However, we believe that to this bias must be added the immortal-time bias. This bias, combined with healthy-vaccinee bias, leads to mortality results that are grossly devoid of biological meaning.

We also reiterate that our various contributions to the scientific debate have never required corrections to the articles we commented on, as Manzoli et al. claim. Instead, we aim to provide constructive criticism to the scientific debate on issues of great relevance to health policy. We hope for an open, evidence-based debate, free from methodological errors and censorship, provided that the scientific validity of the data and methods used is safeguarded.

If Acuti Martellucci et al. (2025[1]) make available the datasets used for their analysis, as already requested in our letter, we will be able to verify the method used to avoid immortal time bias and compare it with our approach.

## Declaration

### Conflict of interest

The authors have nothing to declare.

### Artificial Intelligence (AI) - assisted technology

We did not use artificial intelligence.
